# DNA methylation at modifier genes of lung disease severity is altered in cystic fibrosis

**DOI:** 10.1186/s13148-016-0300-8

**Published:** 2017-02-14

**Authors:** Milena Magalhães, Isabelle Rivals, Mireille Claustres, Jessica Varilh, Mélodie Thomasset, Anne Bergougnoux, Laurent Mely, Sylvie Leroy, Harriet Corvol, Loïc Guillot, Marlène Murris, Emmanuelle Beyne, Davide Caimmi, Isabelle Vachier, Raphaël Chiron, Albertina De Sario

**Affiliations:** 1 Laboratoire de Génétique de Maladies Rares, EA7402 Montpellier University, Montpellier, France; 2 Equipe de Statistique Appliquée—ESPCI ParisTech, PSL Research University—UMRS1158, Paris, France; 3 Laboratoire de Génétique Moléculaire—CHU Montpellier, Montpellier, France; 4 CRCM, Renée Sabran Hospital—CHU Lyon, Hyères, France; 5 CRCM, Pasteur Hospital—CHU Nice, Nice, France; 6 Sorbonne Universités, UPMC Univ Paris 06, Paris, France; 7 INSERM U938—CRSA, Paris, France; 8 APHP, Trousseau Hospital, Paris, France; 9 CRCM, Larrey Hospital—CHU Toulouse, Toulouse, France; 10 CRCM, Arnaud de Villeneuve Hospital—CHU Montpellier, Montpellier, France

**Keywords:** DNA methylation, Co-methylation, Nasal epithelial cells, Cystic fibrosis, Modifier gene, Pulmonary function, Polymorphism, Next-generation sequencing, Pyrosequencing

## Abstract

**Background:**

Lung disease progression is variable among cystic fibrosis (CF) patients and depends on DNA mutations in the *CFTR* gene, polymorphic variations in disease modifier genes, and environmental exposure. The contribution of genetic factors has been extensively investigated, whereas the mechanism whereby environmental factors modulate the lung disease is unknown. In this project, we hypothesized that (i) reiterative stress alters the epigenome in CF-affected tissues and (ii) DNA methylation variations at disease modifier genes modulate the lung function in CF patients.

**Results:**

We profiled DNA methylation at *CFTR*, the disease-causing gene, and at 13 lung modifier genes in nasal epithelial cells and whole blood samples from 48 CF patients and 24 healthy controls. CF patients homozygous for the p.Phe508del mutation and ≥18-year-old were stratified according to the lung disease severity. DNA methylation was measured by bisulfite and next-generation sequencing. The DNA methylation profile allowed us to correctly classify 75% of the subjects, thus providing a CF-specific molecular signature. Moreover, in CF patients, DNA methylation at specific genes was highly correlated in the same tissue sample. We suggest that gene methylation in CF cells may be co-regulated by disease-specific *trans*-factors. Three genes were differentially methylated in CF patients compared with controls and/or in groups of pulmonary severity: *HMOX1* and *GSTM3* in nasal epithelial samples; *HMOX1* and *EDNRA* in blood samples. The association between pulmonary severity and DNA methylation at *EDNRA* was confirmed in blood samples from an independent set of CF patients. Also, lower DNA methylation levels at *GSTM3* were associated with the GSTM3*B allele, a polymorphic 3-bp deletion that has a protective effect in cystic fibrosis.

**Conclusions:**

DNA methylation levels are altered in nasal epithelial and blood cell samples from CF patients. Analysis of *CFTR* and 13 lung disease modifier genes shows DNA methylation changes of small magnitude: some of them are a consequence of the disease; other changes may result in small expression variations that collectively modulate the lung disease severity.

**Electronic supplementary material:**

The online version of this article (doi:10.1186/s13148-016-0300-8) contains supplementary material, which is available to authorized users.

## Background

Environmental factors (i.e., nutrition, maternal diet, pollution, exercise, and lifestyle) influence the phenotype of living organisms by shaping their epigenome and consequently by affecting gene expression [1]. Change in the epigenome could contribute to human diseases and might explain the incomplete penetrance of some mutations as well as the age of appearance of symptoms [2].

Cystic fibrosis (CF) is a monogenic disease that results from mutations in the *cystic fibrosis transmembrane conductance regulator* (*CFTR*) gene that encodes a cAMP-regulated epithelial chloride channel. This life-threatening disease is characterized by recurrent pulmonary infections, chronic inflammation, pancreatic insufficiency, and male infertility. Although multiple organs are affected, morbidity and mortality are mainly due to the lung disease because chronic infections and abnormal inflammation lead to progressive airway destruction. Lung disease progression is variable among CF patients and depends on the combination of three factors: (i) DNA mutations in the *CFTR* gene, (ii) polymorphic variations in other genes, and (iii) environmental exposure.

The contribution of genetic factors to CF phenotype has been extensively investigated by previous studies [3]. DNA mutations have been classified in six groups, depending on the mechanism by which they alter CFTR synthesis, traffic, and function [4]. The p.Phe508del mutation (deletion of the phenylalanine residue at position 508) leads to protein misfolding and degradation. This mutation is very frequent in the Caucasian population (it is homozygous in 40% of CF patients) and is generally, but not always, associated with a severe phenotype. Genetic and transcriptomic studies have provided a rich compilation of genes that can modify the CF outcome and are responsible for the disease variability [5–7]. Genotype-phenotype correlations in CF twins showed that environmental factors also contribute to pulmonary function variation in CF patients [3, 8], but the precise mechanism whereby these factors modulate the lung disease is unknown. The respiratory system is exposed to environmental stimuli (e.g., chemicals, dust, bacteria, or viruses). Of note, CF airway tissues are exposed not only to these external pollutants but also to the high cellular stress generated by the inflammatory and immune responses. Oxidative products generated by the inflammatory response can alter DNA methylation in both directions. Oxidation of 5-methylcytosines and 8-guanosines hinders MBP and DNMT1 binding, favoring loss of DNA methylation [9]. Oxidative compounds produced by the neutrophilic response generate halogenated cytosines that, because they mimic CpG methylation, are recognized by the methyl-binding proteins (MBP) and by the DNMT1 and, hence, favor methylation gain [10, 11]. In CF airway tissues, the oxidative stress is high and the neutrophil response particularly strong. Therefore, we hypothesized that (i) reiterative stress alters the epigenome in CF-affected tissues and (ii) DNA methylation changes at CF modifier genes contribute to the lung function variations observed in CF patients. To test our hypotheses, we profiled DNA methylation in healthy controls and homozygous p.Phe508del CF patients stratified according to their pulmonary function. We analyzed *CFTR*, the disease-causing gene, and 13 lung modifier genes. Ten genes were identified by genetic association studies. They encode proteins involved in inflammatory and immune responses (*TLR2*, *TLR5*, *TGFβ2*, and *IFRD1*), oxidative stress (*HMOX1*, *GSTM1*, and *GSTM3*), bronchoconstriction (*EDNRA*), and mucus structure and hydration (*MUC5AC* and *ENaCγ*). Three genes (*ATF1*, *DUOX2*, and *YY1)* were differentially expressed in nasal epithelial cells collected from CF patients characterized by extreme disease phenotypes [5].

A major hurdle when addressing the epigenome effects on disease severity is to gather appropriate tissue samples from the patients. Here, we used nasal epithelial cells (NEC), which are an informative model to study DNA methylation in airway diseases [12], and blood cells because most of the analyzed genes encode proteins that are involved in the inflammatory and immune responses.

## Results

### DNA methylation analysis in NEC and blood samples

The study was carried out in NEC and blood samples from the METHYLCF cohort that includes 48 CF patients and 24 healthy controls (Table 1). Using bisulfite and next-generation sequencing (BS-NGS), we analyzed DNA methylation at CpG islands associated with *CFTR* and 13 CF lung modifier genes (Table 2). The analyzed regions ranged from 133 to 264 bp, included from 5 to 26 CpG dinucleotides, and were less than 1550 bp away from the transcriptional start site (TSS), except for the *MUC5AC* CpG island that was in the gene body (from exon 35 to intron 35–36). In each region, we measured the methylation at single CpG dinucleotides and the mean DNA methylation. DNA methylation was profiled in 72 blood samples (all patients and controls) and in 63 NEC samples (39 CF patients and all healthy controls). Six patient NEC samples did not provide enough genomic DNA and three samples were reserved for further analyses. To evaluate the repeatability of the BS-NGS methylation analyses, we duplicated the measurements of the 14 genes in four CF patients in both tissues. The estimated standard deviation of DNA methylation reached 0.44 in the logit unit used for variance homogenization. This is quite high because the standard deviation of the methylation around its mean value was of the order of 0.43 in blood and of 0.52 in NEC samples, for both the CF patients and the healthy controls. Nevertheless, the correlation between DNA methylation data in the two independent experiments was excellent (Spearman’s *r* = 0.97, *p* = 0) (Additional file 1: Figure S1).

**Table 1:** Demographic and relevant clinical features of CF patients and controls

	Discovery set (METHYLCF)					Replication set (FrGMC)		
	C (*n* = 24)	CF (*n* = 48)	Mild (*n* = 23)	Intermediary (*n* = 13)	Severe (*n* = 12)	CF (*n* = 30)	Mild (*n* = 18)	Severe (*n* = 12)
Age, year^a^	37	26	34	25.5	22	24.5	27.0	23.5
Sex, M:F	13:11	32:16	17:6	11:2	4:8	19:11	14:4	5:7
BMI (kg/m^2^)^a^		20.9	22.1	20.5	19.8	19.9	21.4	18.0
Weight (kg)^a^		60.0	62.0	60.0	52.0	56.0	60.0	45.5
Height (cm)^a^		170	170	171	168	168.0	170.0	161.5
FEV_1_%^a^		48.0	64.8	48.0	41.5	91.0	102.0	24.0
FVC %^a^		74.0	87.0	67.0	66.5	98.5	105.0	46.0
% PI		100	100	100	100			
% Diabetes		36.7	47.8	23.0	33.3			
% Atopy		28.3	31.8	23.1	27.3			
% *P. Aeruginosa*		93.9	91.3	100	90.9			
% MRSA		34.1	22.7	30.8	60.0			
% *Aspergillus*		18.2	26.1	14.3	20.0			

*PI* pancreatic insufficiency

^a^Median

**Table 2:** *CFTR* and CF modifier genes

Gene symbol	Gene name	Genomic coordinates^a^	nb. CpG^b^	Amplicon size (bp)	Differentially methylated CpG sites^c^	
					Blood	NEC
*ATF1*	Activating transcription factor1	chr12:50,764,850-50,765,098	12	249		
*CFTR*	Cystic fibrosis transmembrane conductance regulator	chr7:117,479,627-117,479,759	10	133	1(−)	
*DUOX2*	Dual oxidase 2	chr15:45,114,541-45,114,722	11	182		
*EDNRA*	Endothelin receptor type A	chr4:147,480,957-47,481,216	21	260	2(–) 3(–) 4(–) 8(–) 9(–) 16(–)	5(+) 10(+)
*ENaCγ*	Epithelial sodium channel	chr16:23,182,420-23,182,665	23	246	2(–) 9(–) 11(+)	2(–) 6(+) 16(–)
*GSTM1*	Glutathione S-transferase mu 1	chr1:109,687,687-109,687,897	13	211		
*GSTM3*	Glutathione S-transferase mu 3	chr1:109,740,573-109,740,793	9	221	1(–) 3(–) 4(–) 5(–) 6(–) 7(–) 8(–)	4(–) 9(–)
*HMOX1*	Heme oxygenase 1	chr22:35,381,269-35,381,436	5	168	2(–) 3(–) 4(+) 5(–)	2(+)
*IFRD1*	Interferon-related developmental regulator 1	chr7:112,450,883-112,451,040	12	158		10(–)
*MUC5AC*	Mucine 5 AC	chr11:1,194,622-1,194,807	13	186	1(+) 10(+) 12(+) 13(–)	1(+) 3(+) 4(+) 5(–) 8(+) 10(+) 11(+) 12(+) 13(+)
*TGFβ*	TGFβ1 Transforming growth factor	chr19:41,353,542-41,353,740	19	199		10(–)
*TLR2*	Toll-like receptor 2	chr4:153,684,576-153,684,704	12	129	8(–)	
*TLR5*	Toll-like receptor 5	chr1:223,142,813-223,142,967	8	155	8(–)	
*YY1*	Yin-Yang 1 transcription factor	chr14:100,240,497-100,240,751	26	255	8(–) 22(–)	

^a^Human Genome GRCh38/hg38 build

^b^nb of CpG in the analyzed region

^c^Position of the CpG in the analyzed sequence. Plus signs mean hypermethylated and minus signs mean hypomethylated CpG in CF patients

**Fig. 1:**
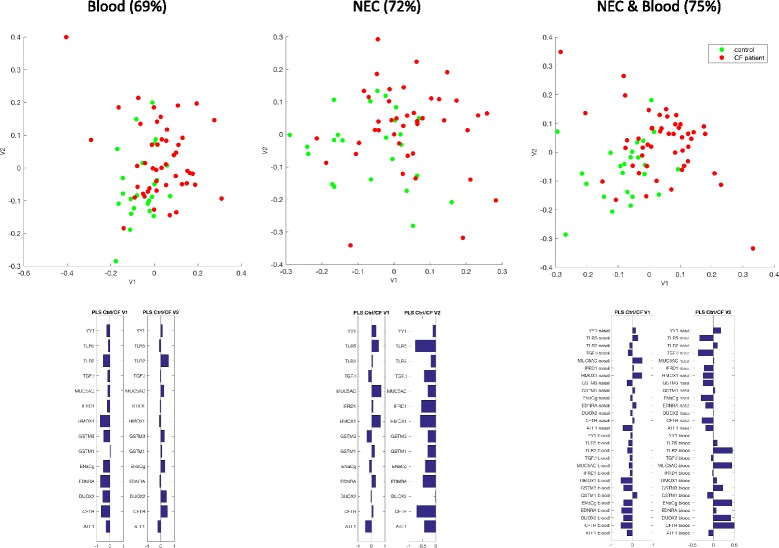
(*Top*) Partial least square (PLS) discriminant analyses of CF patients and controls in blood, NEC, and both tissue samples and the percentage of correct classification of the subjects in each analysis. *v1*,*v2* are the scores on the first two PLS axes. (*Bottom*) The descriptors are mean DNA methylation of 14 genes

In the control samples, DNA methylation was very high at *MUC5AC* (median value 95% in blood and 83% in NEC samples), high at *TLR5* (median value 38% in blood and 26% in NEC samples), and low in the other genes (<20% for both sample types) (Additional file 2: Figure S2). A partial least square discriminant analysis of the mean DNA methylation (the descriptors were the percentage of DNA methylation at the 14 genes in blood and NEC samples) provided 75% of correct classification of CF patients versus controls (Fig. 1). The percentage of correct classification was slightly lower when we used DNA methylation data from NEC samples alone (72%) and even lower with data from blood samples (69%) (Fig. 1).

Besides the mean DNA methylation, we calculated the DNA methylation at individual CpG dinucleotides (*n* = 194 in the fourteen genes). Forty-two CpG sites (21%) in nine genes were differentially methylated between patients and controls in at least one tissue (Table 2). Specifically, 19 CpG sites were differentially methylated in NEC samples and 29 in blood samples. In NEC samples, most CpG sites were more methylated in CF patients than in controls (12 out of 19). Conversely, in blood samples, most of the differentially methylated CpG sites (24 out of 29) were less methylated in CF patients than in controls.

### DNA methylation correlations in CF cells

**Fig. 2:**
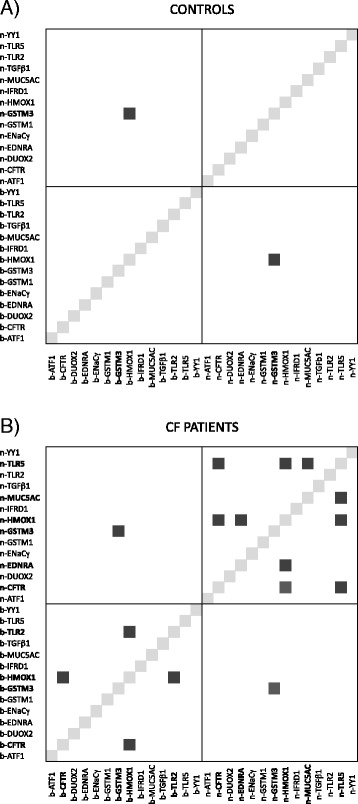
The matrices show inter-tissue (mean DNA methylation of a gene in both cell types) and intra-tissue (mean DNA methylation of two genes in one tissue) correlations in controls (**a**) and CF patients (**b**). The prefix n- or b- in front of the gene name indicates that DNA methylation was measured in NEC or blood samples, respectively. Significant correlations (*black square*) were calculated using Spearman’s test with a Bonferroni-controlled family-wise error rate (FWER) = 10%

Next, we looked for inter-tissue (DNA methylation of a gene in both cell types) and intra-tissue (DNA methylation of two genes in the same tissue) correlations. Data from CF patients and controls were analyzed separately using stringent criteria (Bonferroni-controlled family-wise error rate (FWER) = 10%). Correlations were calculated using the mean DNA methylation of each gene region. Interestingly, DNA methylation at *GSTM3* was highly correlated in NEC and blood samples collected from the same individuals, both in controls and CF patients (Fig. 2). This finding suggests that methylation level at *GSTM3* is under genetic control.

Moreover, a few intra-tissue correlations were found in genomic DNA from CF patients (Fig. 2). Specifically, in NEC samples, we found two co-methylation modules: (i) the DNA methylation level of *TLR5* correlated with that of *MUC5AC*, *CFTR*, and *HMOX1* and (ii) the DNA methylation level of *HMOX1* correlated with that of *EDNRA* and *CFTR*. In blood samples, the DNA methylation level of *HMOX1* correlated with that of *CFTR* and with *TLR2*.

In control samples, no intra-tissue correlations were significant with a FWER of 10%. All genes were expressed in the tissue where their co-methylation was found, except for *CFTR* that was not expressed in blood samples. Thus, gene expression does not seem to be an essential pre-requisite for co-methylation. We assessed gene expression by RT-PCR using NEC and blood mRNAs from two healthy individuals (data not shown).

### *HMOX1* was differentially methylated in NEC and blood samples from CF patients

**Fig. 3:**
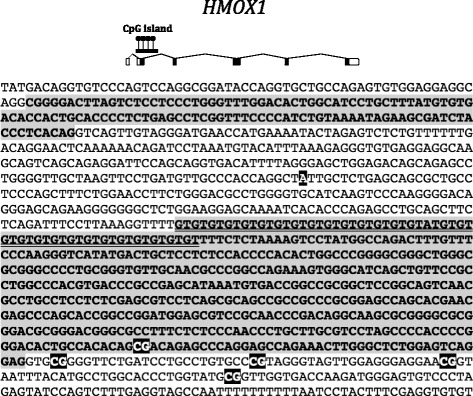
*Top*, *HMOX1* exon-intron structure and position of the CpG island. *Bottom*, *HMOX1* partial genomic sequence showing exons 1 and 2 (*gray background*) and introns (*white background*). Also shown, the five CpG (in *white* on *black* background) where we measured DNA methylation, the major “A” allele of SNP rs2071746 (*white on black background*) and the polymorphic (GT)_*n*_ microsatellite (*underlined*) in exon 2

**Fig. 4:**
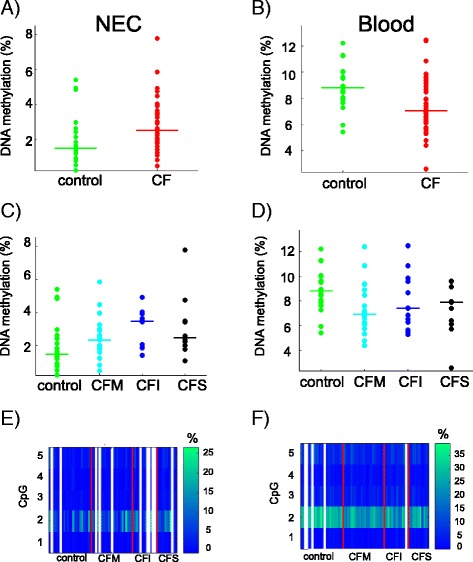
DNA methylation at *HMOX1*. The *dot plots* represent the mean DNA methylation of *HMOX1* in CF patients compared with controls in NEC (Student *p* = 0.018) (**a**) and in blood (Wilcoxon *p* = 0.009) samples (**b**). DNA methylation levels depended on pulmonary severity (NEC, ANOVA *p* = 0.052 (**c**); blood, Kruskal-Wallis *p* = 0.035 (**d**)). The *horizontal line* indicates the median in each group. The heat maps represent the DNA methylation at five CpG dinucleotides in NEC (**e**) and blood (**f**) samples. *White lines* represent missing data. *CFM* mild CF patient, *CFI* intermediary CF patient, *CFS* severe CF patient

Next, we focused on genes that were differentially methylated in CF patients or groups of patients. *HMOX1* was previously identified as a CF modifier gene by genetic association studies [13]. We measured DNA methylation at the CpG island that overlaps exon 2 (Fig. 3). Using the mean DNA methylation in the region, we found that *HMOX1* was differentially methylated in NEC samples (Student *p* = 0.018) and blood cell samples (Wilcoxon *p* = 0.009) of CF patients compared with controls, but the direction of the methylation change was not the same in the two tissue models (Fig. 4a, b). Moreover, DNA methylation was associated with lung disease severity (ANOVA *p* = 0.052 in NEC samples; Kruskal-Wallis *p* = 0.035 in blood samples) (Fig. 4c, d). One CpG dinucleotide (CpG#2) was more methylated than the other four CpG in the region (approximately 30% compared with <10%) (Fig. 4e, f). CpG#2 was differentially methylated in CF patients compared with controls in both tissues (Bonferroni corrected *q* = 0 in blood and *q* = 2.7 10^−3^ in NEC) and was associated with pulmonary severity in blood samples (Kruskal-Wallis *p* = 0.0019).

### DNA methylation at *HMOX1* was not associated with nearby polymorphisms

Previous studies showed that two polymorphic sequences in the 5′ untranslated region of *HMOX1* were associated with lung function in airway diseases. Specifically, the minor allele of the A(-413)T variant (rs2071746) was associated with CF lung disease severity in two independent cohorts [13] (Fig. 3). Next to this single-nucleotide polymorphism (SNP), the length of a (GT)_*n*_ microsatellite correlated with pulmonary severity in airway (emphysema and COPD) and cardiovascular diseases [14, 15] (Fig. 3). Long microsatellites (>32 repeats) were associated with lower levels of transcription in vitro and with an adverse clinical phenotype in patients [16]. Because these polymorphisms were close (600 bp upstream) to the region analyzed in this study, we asked whether they affected DNA methylation. We assessed the A(-413)T SNP and the microsatellite length in CF patients and healthy volunteers of the METHYLCF cohort and found that DNA methylation levels (mean methylation of the amplicon and methylation at CpG#2) in NEC and blood samples did not correlate with any genotype (Spearman’s correlation test) (Additional file 3: Table S2).

### DNA methylation at *HMOX1* was not associated with a significant change of gene expression

**Fig. 5:**
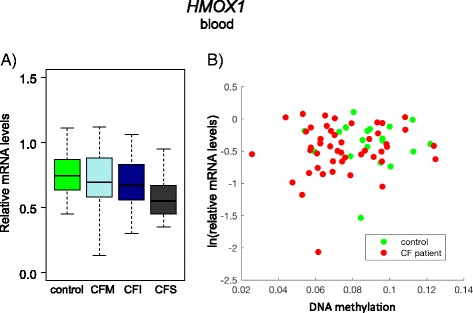
*HMOX1* gene expression in blood samples. **a** The *box plots* represent the relative expression of *HMOX1* in CF patients with different lung disease and controls (Kruskal-Wallis *p* = 0.39). *CFM* mild CF patient, *CFI* intermediary CF patient, *CFS* severe CF patient. **b** Correlation between gene expression and mean DNA methylation levels in blood samples from CF patients and controls. Whole cohort (Spearman’s *r* = 0.09, *p* = 0.48); controls (Spearman’s *r* = −0.14, *p* = 0.62); CF patients (Spearman’s *r* = 0.08, *p* = 0.58)

Next, we asked whether DNA methylation at *HMOX1* affected gene expression. In blood cells, DNA methylation and gene expression were analyzed in samples collected from the same individuals. *HMOX1* was not differentially expressed in CF patients compared with controls (Wilcoxon *p* = 0.11) or in patients stratified according to the lung disease severity (Kruskal-Wallis *p* = 0.39) (Fig. 5a). Also, expression and DNA methylation levels (mean methylation of the amplicon and methylation at CpG#2) were not correlated, be it in the whole cohort (Spearman’s *r* = 0.09, *p* = 0.48) or separately in the control (Spearman’s *r* = −0.14, *p* = 0.62) and in the CF (Spearman’s *r* = 0.08, *p* = 0.58) populations (Fig. 5b).

RNA could not be extracted from the NEC samples of the METHYLCF cohort because the whole amount of cells had to be used to isolate genomic DNA. Therefore, to determine the expression levels in NEC samples, we inspected data from three publicly available transcriptomic studies [5, 17, 18]. *HMOX1* was not differentially expressed in CF compared with control NEC. The only study that compared mild versus severe CF patients was not informative for this gene [5].

### *EDNRA* was differentially methylated but not differentially expressed in CF blood samples

**Fig. 6:**
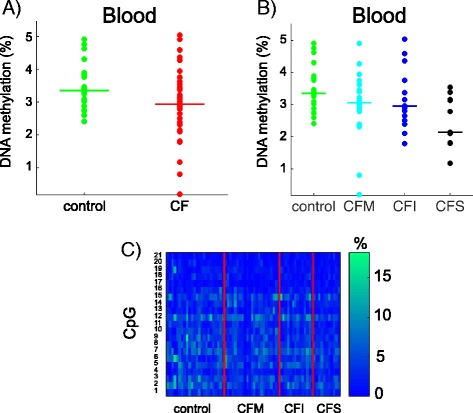
DNA methylation at *EDNRA* in blood samples*.* **a** The dot plots represent the mean DNA methylation of *EDNRA* in CF patients compared with controls (Wilcoxon *p* = 0.017). **b** DNA methylation at *EDNRA* correlated with pulmonary severity (Kruskal-Wallis *p* = 0.028). The *horizontal line* indicates the median in each group. **c** The heat map represents DNA methylation at 21 CpG dinucleotides. *CFM* mild CF patient, *CFI* intermediary CF patient, *CFS* severe CF patient

*EDNRA* encodes a G protein-coupled receptor that, following binding to endothelin, triggers cellular proliferation and contraction of smooth muscle cells. In CF airways, higher level of endothelin may contribute to the pulmonary phenotype [19]. In the METHYLCF cohort, *EDNRA* was less methylated in CF than in control blood samples (Wilcoxon *p* = 0.017) and DNA methylation level correlated with the lung disease severity (Kruskal-Wallis *p* = 0.028) (Fig. 6a, b). The DNA methylation at individual CpG sites was homogeneous, close to the mean methylation in the region (Fig. 6c).

Gene expression was not detectable in blood cells, even in CF samples where *EDNRA* was less methylated. Thus, we concluded that loss of DNA methylation at *EDNRA* was a consequence rather than a cause of lung disease severity.

### DNA methylation levels at *GSTM3* were associated with lung disease severity and correlated with the GSTM3*B allele

**Fig. 7:**
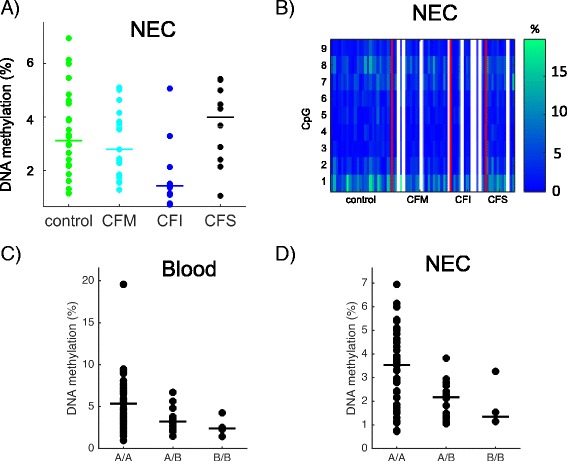
DNA methylation at *GSTM3.* The mean DNA methylation at *GSTM3* depended on pulmonary severity (ANOVA *p* = 0.016) (**a**). The *heat maps* represent DNA methylation at nine CpG dinucleotides in NEC samples; *white lines* represent missing data (**b**). Low methylation levels correlated with the GSTM3*B allele both in blood (Spearman’s *r* = −0.42, *p* = 3 10^−4^) (**c**) and NEC samples (Spearman’s *r* =−0.43, *p* = 5 10^−4^) (**d**). *CFM* mild CF patient, *CFI* intermediary CF patient, *CFS* severe CF patient. The *horizontal line* in the *dot plots* indicates the median in each group. *A/A* homozygous GSTM3*A, *A/B* heterozygous, *B/B* homozygous GSTM3*B

The mean DNA methylation at *GSTM3* was not significantly different in CF and control samples; however, it was associated with CF lung disease severity in NEC samples (ANOVA *p* = 0.016) (Fig. 7a). DNA methylation at individual CpG sites was homogeneous (Fig. 7b).

Previous studies showed that various polymorphisms of the *GST(M)* genes contribute to lung disease severity in CF patients [20] and that GST activity may modulate *P. aeruginosa* lung infection [21]. Of note, the GSTM3*B allele, a 3-bp deletion that has a protective effect in CF patients, is 6.1 kb downstream of the region analyzed in this study. To determine whether this polymorphic sequence affected DNA methylation levels, we genotyped patients and controls for the micro-deletion (Additional file 3: Table S2). Interestingly, in both NEC and blood samples, DNA methylation levels at *GSTM3* correlated with the presence of the GSTM3*B allele (Spearman’s NEC *r* = −0.43 *p* = 5 10^−4^; blood *r* = −0.42 *p* = 2.8 10^-4^). DNA methylation levels in homozygous GSTM3*B carriers were lower than in heterozygous carriers, where they were lower than in homozygous GSTM3*A carriers (Fig. 7c, d).

### Replication of DNA methylation analysis in an independent set of CF patients

To replicate data obtained in the METHYLCF cohort, we selected 30 additional p.Phe508del homozygous patients with severe (*n* = 12) or mild lung disease (*n* = 18) from an independent CF cohort enrolled by the French CF Gene Modifier Consortium (FrGMC) [22]. Of note, the phenotype of this set of patients was more extreme than that of the METHYLCF cohort (Table 1). Genomic DNA being available for blood and not for NEC cells, we decided to replicate blood differentially methylated regions (*EDNRA* and *HMOX1*), leaving replication of NEC regions for future studies. DNA methylation was measured by locus-specific pyrosequencing. To analyze *EDNRA*, we used a pyrosequencing assay located 350 bp downstream of the region that was targeted by BS-NGS. In the replication set of patients, DNA methylation at *EDNRA* was significantly associated with lung disease severity (Kruskal-Wallis *p* = 0.047) (Additional file 4: Figure S3). DNA methylation in mild CF patients was higher than in controls (Wilcoxon *p* = 0.023) and slightly higher than in severe patients (not significant). Overall, *EDNRA* DNA methylation levels by pyrosequencing were higher than those obtained by BS-NGS: this is consistent with previous results by Potapova et al. [23] who compared the two methods and showed a trend towards higher values in the range between 0 and 20% DNA methylation.

For *HMOX1*, all tested primers failed to provide a linear pyrosequencing signal in the region of interest.

## Discussion

In this study, we provide the first DNA methylation profile using tissue samples collected from CF patients. We measured DNA methylation at CpG islands associated with *CFTR* and 13 CF modifier genes. DNA methylation levels were altered not only in NEC, which are directly affected by the disease (CF patients often have rhinitis and nasal polyposis), but also in blood cells where *CFTR* is not expressed. By combining the DNA methylation data obtained in NEC and blood cells, we correctly classified 75% of the subjects, distinguishing homozygous p.Phe508del CF patients from controls. This finding suggests that DNA methylation variations in specific genes may provide a CF-specific molecular signature.

Our study has also disclosed a number of genes whose methylation seemed to be co-regulated in CF samples. Concomitant DNA methylation changes in two or more genes have been already described in solid tumors, including in lung adenocarcinomas [24] and in sputum samples of asthmatic smokers [25]. More recently, van Eijk *et al*. identified networks of co-methylation and co-expression modules in blood samples collected from healthy individuals [26]. In this genome-wide analysis, co-methylation and co-expression modules contained few overlapping genes, but several pairs of methylation and expression modules were significantly correlated [26]. Moreover, because they were enriched in gene ontology categories, these modules were considered biologically relevant. The actual mechanism responsible for their generation is unknown, however, the existence of factors that affect DNA methylation and gene expression acting in *trans* at the module level was hypothesized [26]. In our study, using stringent conditions, we observed gene co-methylation exclusively in patient samples. Therefore, we suggest the involvement of *trans*-acting factors that are specifically activated by the disease, namely by the oxidative stress and the inflammatory and immune responses. A genome-wide DNA methylation analysis of CF samples is required to better understand this phenomenon.

By comparing patients and controls, we found significant DNA methylation variations at two CF modifier genes: *HMOX1* (in NEC and blood cells) and *EDNRA* (in blood cells). Moreover, the DNA methylation level at three genes (*GSTM3* in NEC and *HMOX1* and *EDNRA* in blood samples) was associated with lung disease severity. The association between pulmonary severity and DNA methylation at *EDNRA* was replicated using blood samples from an independent set of CF patients. The magnitude of the methylation changes in lung severity modifier genes was small. Three lines of evidence show that small epigenomic changes can be biological meaningful. First, many epidemiological studies showed that the environment induces small epigenetic changes associated with a clinical outcome. In patients affected by chronic obstructive pulmonary disease and exposed to fine particulate matter (PM_2.5_) constituents, hypomethylation of the *NOS2A* gene (about −1.5%) was associated with a higher (about +18%) fractional concentration of exhaled nitric oxide (FeNO), a biomarker of airway inflammation [27]. In patients with type 2 diabetes mellitus (T2DM), a CpG dinucleotide in the first intron of the *FTO* gene was hypomethylated (−3.35%) and the odds of belonging to the T2DM group increased by 6.1% for every 1% decrease in DNA methylation [28]. Second, experimental studies in animals showed the impact of small methylation changes on gene expression. In the offspring of rat fed with a protein-restricted diet during pregnancy, a small decrease of DNA methylation in the promoter of *PPARα* was associated with an increase of gene expression [29]. Third, a genome-wide expression analysis in patients affected by type 2 diabetes mellitus showed that small expression changes in multiple genes belonging to the same pathway had a bigger impact than a high-fold change in a single gene [30, 31]. Collectively, these findings lead us to suggest that small DNA methylation variations in lung modifier genes can impact cystic fibrosis severity.

*HMOX1* encodes a protein that is important for iron homeostasis and cell protection from oxidative damage during stress. Activating and repressive factors regulate the *HMOX1* basal expression by interacting with the promoter and various stimuli (i.e., heme, cadmium, and oxidative stress) switch on its induced expression via binding to responsive elements [32]. Of note, the CpG island targeted in our DNA methylation analysis contains an *HMOX1* hydrogen peroxide-responsive element [14]. CF tissues are exposed to continuous stress by the immune and inflammatory responses. Here, we found that *HMOX1* was differentially methylated both in blood and NEC samples from CF patients compared with controls, but the direction of the methylation change was not the same in the two tissue models. One possible explanation is that DNA methylation levels result from a balance between the burden of halogenic compounds produced by the inflammatory response (especially by neutrophils) that favors methylation gain [10, 11] and other oxidative products responsible for methylation loss [9]. The contribution of these opposing factors is likely to be different in blood and NEC because NEC are directly affected by cystic fibrosis. In addition, in NEC samples, the increase of DNA methylation at the promoter of *HMOX1* was non-monotonic in CF patients stratified according to the lung disease severity. The intensity of the inflammatory response and of the oxidative stress in the airway tissues varies among patients and correlates with the lung disease [33, 34]. The proportion of oxidative products changing DNA methylation in opposite directions may be variable in stratified CF patients so that the final ratio results in a U-shaped curve. The possible effect of DNA methylation on *HMOX1* transcription deserves further analysis. In NEC samples, the small amount of cells did not allow us to carry DNA methylation and gene expression analysis on the same samples. In blood samples, we failed to demonstrate a significant impact of DNA methylation on expression, possibly due to the lack of statistical power of the present cohort.

*EDNRA* encodes a G protein-coupled receptor that, following ligation to endothelin, causes contraction of smooth cells. Previous genetic studies showed an association between *EDNRA* DNA polymorphisms and pulmonary disease in four independent cohorts of CF patients [19]. Also, a functional study showed that an allele that is deleterious for the lung function resulted in higher *EDNRA* mRNA levels in human tracheal smooth muscle cells [19]. Our study shows that *EDNRA* was hypomethylated in CF patients and DNA methylation levels were associated with pulmonary disease severity in blood cells. Because *EDNRA* transcripts were not detected in control nor in CF samples, we conclude that loss of DNA methylation had no impact on gene expression and was probably a consequence rather than a cause of lung disease severity.

Compelling evidence shows that DNA methylation is affected not only by environmental but also by genetic factors. Of note, methylation levels at 2–7% of CpG sites are associated with *cis*-DNA variants and may provide the molecular mechanisms for the associated quantitative trait locus [26]. In the present study, we realized that two differentially methylated regions mapped close to polymorphic sequences that have been previously shown to be associated with the pulmonary function in airway diseases: two DNA variants were in the 5′ untranslated region of *HMOX1* and the third one was in the body of *GSTM3*. Since we found no correlation between DNA methylation levels and two polymorphic sequences in *HMOX1*, we suggest that DNA methylation and the two polymorphisms are independently associated with lung function. This result should be validated in an independent cohort. Conversely, two findings in our study suggest that DNA methylation in the *GSTM3* gene is under genetic control. First, DNA methylation at *GSTM3* was highly correlated with the presence of the GSTM3*B allele both in NEC and blood samples. Second, we found a high positive correlation between *GSTM3* DNA methylation levels in the blood and NEC samples from the same individuals. These results are consistent with a previous study showing that diplotypes in the *GSTM3* gene predicted DNA methylation levels at five CpG dinucleotides scattered in the gene, outside the region we analyzed [35]. The GSTM3*B allele, a 3-bp deletion in intron 6, is associated with higher level of GSTM3 mRNA and protein expression [36]. To explain this association, it was proposed that the 3-bp deletion generates a binding site for the transcription factor YY1 [37]. We hypothesize that upon activation by YY1 or another transcription factor, the GSTM3*B intronic sequence binds to the gene promoter via a chromatin loop and causes a reduction in the DNA methylation level in the same region. The GSTM3 protein conjugates various toxic compounds to glutathione, thus, similarly to HMOX1, has a protective effect in cells, and is particularly beneficial to CF damaged tissues.

The present study has limitations. We analyzed 48 CF patients and 24 healthy controls. Confirmatory studies should be carried out on a larger number of patients. DNA methylation was analyzed in 14 lung modifier genes and restricted to the promoter regions. Future studies should cover the whole genome including other genic and intergenic regulatory regions (enhancers, insulators, etc). We could not analyze gene expression in NEC samples because the whole amount of cells had to be used for DNA extraction.

## Conclusions

In summary, we showed that DNA methylation was altered in nasal epithelial and blood samples from CF patients and, using stringent conditions, we observed modules of gene co-methylation exclusively in patient samples. Through the analysis of 13 lung disease-modifiers genes, we found DNA methylation changes of small magnitude in two genes (*HMOX1* and *EDNRA*). DNA methylation was associated with pulmonary severity in three genes (*HMOX1*, *GSTM3*, and *EDNRA)* and with a polymorphic deletion that has a protective effect in cystic fibrosis at one gene *(GSTM3)*. Some of these small DNA methylation changes are a consequence of the disease. Other changes may result in small expression variations that collectively and over time modulate the lung disease severity. Genome-wide epigenomic, transcriptomic and genomic analyses are needed to further understand how genetic and epigenetic factors contribute to the large spectrum of lung disease severity in cystic fibrosis.

## Methods

### Study cohorts

The study was approved by the local Institutional Review Board (CPP Sud Méditerranée III, Nîmes #2013.02.01bis). Informed written consent was obtained from all participants. Table 1 lists the demographic and relevant clinical features of two cohorts. CF patients were homozygous for the p.Phe508del mutation and ≥18-year-old. Exclusion criteria for CF patients included lung transplantation and pulmonary exacerbation during sample collection.

The *METHYLCF* cohort includes 48 CF patients and 24 healthy controls with no history of airway diseases or allergy. It was enrolled in four CF centers in the South of France. CF patients were stratified into three groups based on the severity of the lung disease and mainly using the FEV_1_% predicted: mild (48% of patients), intermediary (27%), and severe (25%). Patients with FEV_1_% predicted values that corresponded to the top and bottom quartiles were classified as mild and severe, respectively [38]. CF patients of age ≥34 years were considered mild because of their long survival. The age distribution did not differ between patients and controls (Wilcoxon *p* = 0.30). The male-to-female ratio was slightly, but not significantly, higher in CF patients than in controls (*χ* ^2^ *p* = 0.22).

From the already available *FrGMC* cohort (French Ethical Board, CPP #2004/15) [22], a replication set of CF patients (12 patients with severe and 18 patients with mild pulmonary disease) was selected. They were stratified using the same criteria as for the METHYLCF cohort.

### Biological samples

Biological samples were collected from the METHYLCF cohort, whereas blood genomic DNA was already available for the replication FrGMC cohort.

*Nasal epithelial cells* were collected from the inferior turbinate using nasal curettes (Rhino-probe, Arlington) after nebulization with 5% xylocaine (Astrazeneca, France). NEC were collected from both nostrils, pooled together in 1 ml RNA protect Cell Reagent (#76526 Qiagen), and then shipped to the handling center at room temperature.

*Whole blood samples* were collected in EDTA (5 ml) and in PAXgene (2.5 ml) tubes (#762165, Becton Dickinsen) for DNA and RNA extraction, respectively.

### DNA extraction

NEC collected in RNAprotect Cell Reagent (#76526 QIAGEN) were treated with 1 mg/mL RNAse. Genomic DNA was extracted using the QIAamp DNA Micro Kit (#56304, QIAGEN) as previously described [39]. The mean DNA yield was 5.1 ± 2.8 μg in controls and 3.9 ± 3.1 μg in CF patients (range 0 to 12.4 μg). DNA yield was not significantly different between groups (Wilcoxon *p* = 0.19).

Genomic DNA was extracted from whole blood samples using the Flexigene DNA kit (#51206, QIAGEN) according to the manufacturer’s recommendations.

### RNA extraction

RNA was extracted from whole blood samples using the PAXgene Blood RNA kit (#762124, PreAnalytix), according to the manufacturer’s recommendations.

### Bisulfite conversion

NEC and blood DNA samples were treated with sodium bisulfite as previously described [40].

### DNA methylation analysis by amplicon sequencing

Fusion primers were designed to amplify 133 to 264 bp-long amplicons in the region of interest (Additional file 5: Table S1). Each forward primer contained a MID (Multiplex Identifiers, Roche) to allow computational screening of each sample. PCR products were obtained using the PyroMark PCR kit (#978703, QIAGEN), and 10 μM forward and reverse primers in a 25-μl final volume. PCR conditions were 95 °C for 15 min, followed by 94 °C for 30 s, the annealing temperature for 30s, 72 °C for 30 s for 45 cycles, and then 72 °C for 10 min. Amplicons were purified with the QIAquick PCR Purification Kit (#28106 QIAGEN) and quantified using a NanoDrop 2000 Spectrophotometer (Thermo Scientific) and a Qubit 2.0 fluorometer (Life Technologies). In each sequencing run, 112 purified amplicons were pooled in equimolar amounts. Emulsion PCR and subsequent bidirectional sequencing were done according to the GS Junior emPCR Amplification Method Manual-Lib-A (#05996520001, Roche) and GS Junior Sequencing Method Manual (#05996554001, Roche), respectively.

### Sequence analysis

We measured DNA methylation using bisulfite and next-generation sequencing (BS-NGS). To filter and order the raw sequencing data, we developed a pipeline. The script works in a Galaxy environment and includes four steps: (i) a barcode splitter to separate sequences per sample; (ii) a sequence trimming to remove all the MID (multiplex identifiers, Roche) and adaptor sequences; (iii) a barcode splitter to separate sequences per gene; and (iv) analysis of fasta/bam files with BiQAnalyzer HT [41]. BiQAnalyzer HT removes non-fully converted sequences and determines the methylation status of each CpG site within amplicons. It provides a text file where each CpG site is either 1 (methylated) or 0 (unmethylated). A minimal conversion rate of 0.97 was used. Before filtering, the number of reads per analyzed amplicon ranged from 9 to 2704. We retained only the BS-NGS measurements for which the number of sequences was large enough as to have either a coefficient of variation of the mean methylation percentage smaller than 5% or a standard deviation not higher than 1% (the first condition is too stringent for very small methylation percentages). After filtering, 95% of the reads were in the interval [98; 1460].

### DNA methylation analysis by pyrosequencing

PCR products were amplified using the PyroMark PCR Kit ((#978703, QIAGEN) in 25 μL reaction volume. For *EDNRA*, the pool of forward and reverse primers (one of which was biotin-labeled at the 5′) as well as the sequencing primer were from the Hs_EDNRA_02_PM PyroMark CpG Assay (#978746, QIAGEN, Hilden, Germany). The PCR program was 94 °C for 15 min, followed by 94 °C 30 s, 56 °C for 30 s, 72 °C for 30 s during 45 cycles, and 72 °C for 10 min. PCR products were purified using 1 μL Streptavidin Sepharose HP™ (#17-5113-01, GE Healthcare) and a PyroMark Q24 Workstation. Pyrosequencing reactions were performed in a PyroMark Q24 (QIAGEN) using the PyroMark Gold Q24 reagents (#970802, QIAGEN) according to the manufacturer’s instructions. Before the assays, we tested the signal linearity using mixtures of methylated and unmethylated genomic DNA (0, 20, 40, 60, 80, and 100%); standard errors were from three replicates.

### Genotyping

#### HMOX1 (GT)_*n*_ microsatellite

Using blood genomic DNA, we amplified a 113–135-bp DNA fragment spanning the (GT)_*n*_ microsatellite with a FAM-labeled sense primer (5′-AGAGCCTGCAGCTTCTCAGA-3′) and an unlabeled reverse primer (5′-ACAAAGTCTGGCCATAGGAC-3′). The PCR program was 94 °C 30 s, 57 °C 90 s, 72 °C 90 s for 30 cycles. PCR products were analyzed using an ABI 3130xl Genetic Analyzer (Applied Biosystem), and the microsatellite size was measured with the Gene Mapper software (Applied Biosystem).

#### HMOX1 SNP rs2071746

A 139-bp PCR fragment surrounding the A(-413)T SNP (rs2071746) was amplified with the following program: 95 °C 30 s, 64 °C 30 s, 72 °C 30 s for 35 cycles. Primers were forward 5′-GCAGAGGATTCCAGCAGGTG-3′ and reverse 5′-CAGGCGTCCCAGAAGGTTCC-3′. After purification with the QIAquick kit (QIAGEN) and labeling with the Big Dye Terminator (Life Technologies), DNA was sequenced using an ABI 3130xl Genetic Analyzer (Applied Biosystem).

#### GSTM3 *A and GSTM3*B alleles

A 202-bp PCR fragment was amplified using primers 5′-GCTACCTGGACAACTGAAAC-3′ and 5′-CGGTTCTGATCCAAGATATC-3′ and the following program: 95 °C 5 min, then (95 °C 30 s, 56 °C 30 s, 72 °C 1 min) for 25 cycles and 72 °C 15 min. PCR products were analyzed using an ABI 3130xl Genetic Analyzer (Applied Biosystem) and their size measured with the Gene Mapper software (Applied Biosystem).

### Gene expression

For reverse transcription, 500 ng of total blood RNA from each sample was added to Rnase-free water (final volume 8 μl) followed by DNase I treatment for 15 min at room temperature. Samples were then added to a mix containing 4 μl of first strand 5× buffer, 2 μl of 10× dithiothreitol, 1 μl of 10 mM dNTP mix, 300 ng/μl of hexaprimer (random primers), 20–40 U/μl of RNasin® enzyme (Promega), and 200 U/μl of MMLV-RT enzyme (Life Technology). The reverse transcription reaction program consisted of three steps: 10 min at 25 °C, 50 min at 37 °C, and 15 min at 70 °C. mRNA expression was measured using a LightCycler 480 real-time PCR system and SYBR Green I Master mix® (Roche Diagnostics) (primers are listed in Additional file 5: Table S1). Standard curves were generated for each run by serial dilution of control cDNA. Gene expression levels were expressed as ratios relative to that of reference genes (*GAPDH* for *HMOX1* and *TBP* for *EDNRA*). Real-time PCR reactions were done in duplicate in two independent reverse transcriptions.

### Statistical analysis

For a given gene, the mean methylation of each individual site as well as the mean methylation percentage over all sites were left for statistical analysis. To homogenize the variance of the mean methylation percentage (which is maximal at 50% and zero at 0 or 100%), we worked with its logit transformation.

To evaluate the repeatability of the BS-NGS methylation analyses, we duplicated the measurements corresponding to the *n*_g_ = 14 genes of interest for 4 CF patients in the *n*_t_ = 2 tissues (blood and NEC) with 106 degrees of freedom (instead of 4 × *n*_g_ × *n*_t_ = 112 due to few missing values).

To compare the mean methylation level of a given gene in a given tissue between controls and CF patients, and across the whole cohort stratified according to the severity of the lung disease (i.e., controls, mild, intermediary, and severe CF patients), depending on the statistical features of the data (normality or not, homoscedasticity or not), we used either parametric tests (i.e., Student, Welch, and analysis of variance tests) or non-parametric tests (i.e., Wilcoxon and Kruskal-Wallis tests). *P* values <0.05 were considered statistically significant. To compare the methylation status of the individual CpG sites between controls and CF patients, we used Fisher’s exact test. To take the multiplicity of the hypotheses into account, we used Bonferroni’s correction and a family-wise error rate (FWER) of 5% was considered significant.

The ability of the 14 genes in both tissues to discriminate between controls and CF patients was further evaluated using a partial least square discriminant analysis. The descriptors were the normalized mean methylation levels in one of the tissues or both. The PLS response was discrete with two levels, −1 for controls and +1 for CF patients: positive PLS estimates correspond to a classification into the control class and negative ones to a classification into the CF patients class, hence a percentage of correct classification.

We studied the correlations of the mean methylation levels of the genes in both tissues using Spearman’s non-parametric correlation coefficient. To take the multiplicity of the hypotheses into account, we used Bonferroni’s correction and a FWER of 10% was considered significant.

The expression ratios of *HMOX1* in blood obtained with PCR were log transformed before their mean was taken. Because the resulting values were non-Gaussian, the expression levels between controls and stratified or unstratified CF patients were compared with Kruskal-Wallis’ and Wilcoxon’s tests. The correlation with the mean methylation level and the methylation status of the individual CpG sites was analyzed with Spearman’s coefficient.

Spearman’s coefficient was used to test the correlation of lung function (characterized by degree of severity, FEV1% predicted and FVC) with CF patient genotypes at *GSTM3*, (homozygous GSTM3*A, GSTM3*A/GSTM3*B and homozygous GSTM3*B) and at *HMOX1* (rs2071746 A/A, A/T and T/T; and the (GT)_*n*_ microsatellite length where we considered both the largest or the smallest *n* of the two alleles). Multivariate regression models were also used to correct for factors such as demographic and clinical data (Table 1).

## Additional files

Additional file 1: Figure S1. Correlation between DNA methylation data in two independent experiments (Spearman’s *r* = 0.97 *p* = 0). (PNG 113 kb)
Additional file 2: Figure S2. DNA methylation distribution at 14 analyzed genes in CF and control samples. (PDF 212 kb)
Additional file 3: Table S2. Distribution of *HMOX1* and *GSTM3* genotypes in CF patients and controls (DOCX 39 kb)
Additional file 4: Figure S3. DNA methylation analysis at *EDNRA* in an independent set of CF patients. DNA methylation at *EDNRA* was associated with pulmonary severity in blood samples collected from this independent set of severe (CFS) and mild (CFM) CF patients (Kruskal-Wallis *p* = 0.047). CF patients were from the FrGMC cohort; controls were from the METHYLCF cohort. The mean DNA methylation was measured by pyrosequencing. (PNG 72 kb)
Additional file 5: Table S1. Primers. (DOCX 49 kb)

## Abbreviations

BMI: Body mass index
CF: Cystic fibrosis
COPD: Chronic obstructive pulmonary disease
FEV_1_%: Percentage of forced expiratory volume in 1 second
FVC%: Percentage of forced vital capacity
FWER: Family- wise error rate
MRSA: Methicillin-resistant *Staphylococcus aureus*
NEC: Nasal epithelial cells
NGS: Next-generation sequencing
PI: Pancreatic insufficiency
PLS: Partial least square
SNP: Single-nucleotide polymorphism
