# Supplementary figures and images for: DNA methylation at modifier genes of lung disease severity is altered in cystic fibrosis
Source: Clin Epigenetics. 2017 Feb 14;9:19. doi: 10.1186/s13148-016-0300-8 (PMC5310067; doi:10.1186/s13148-016-0300-8)

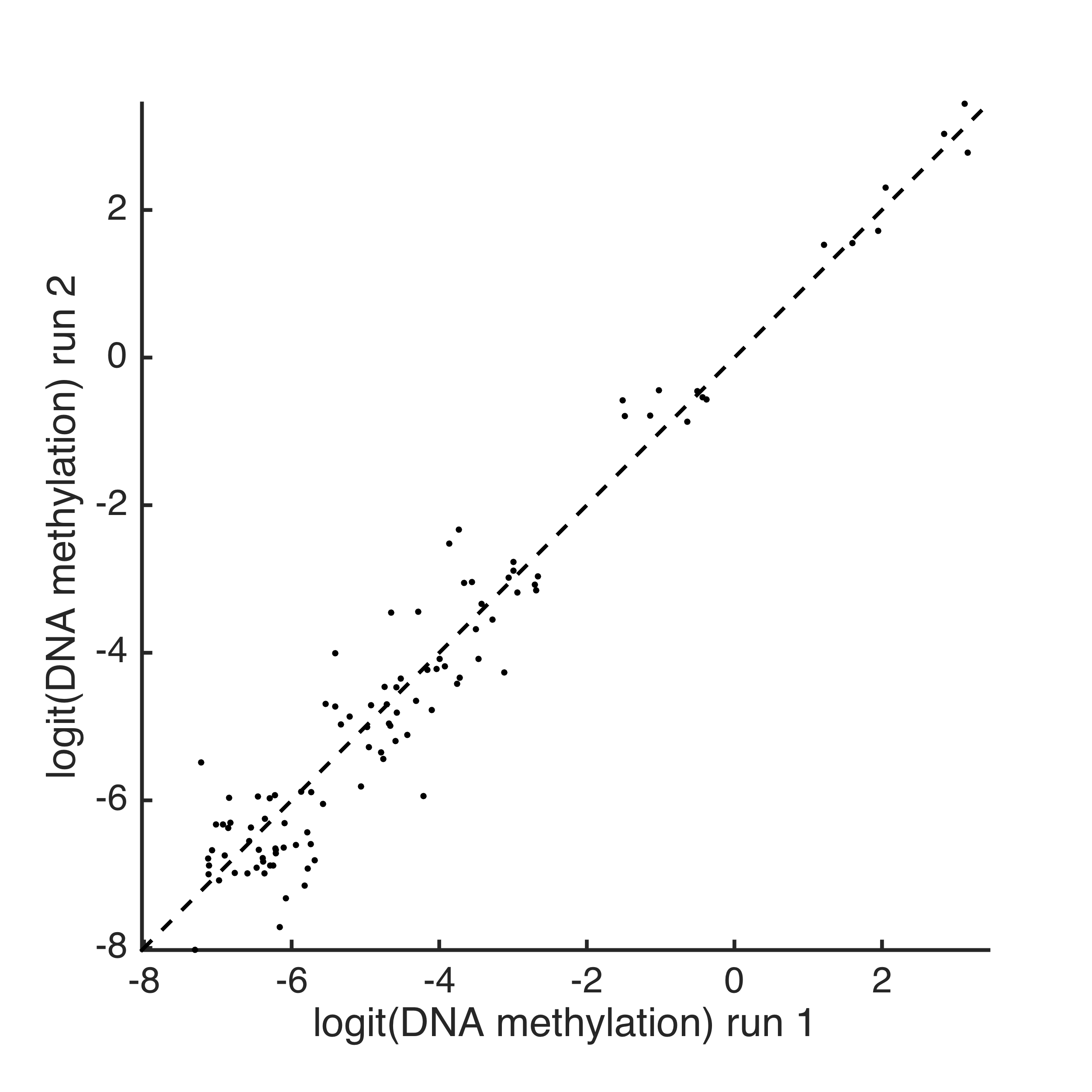

Supplement: Additional file 1: Figure S1. — Correlation between DNA methylation data in two independent experiments (Spearman’s r = 0.97 p = 0). (PNG 113 kb) [file 13148_2016_300_MOESM1_ESM.png]

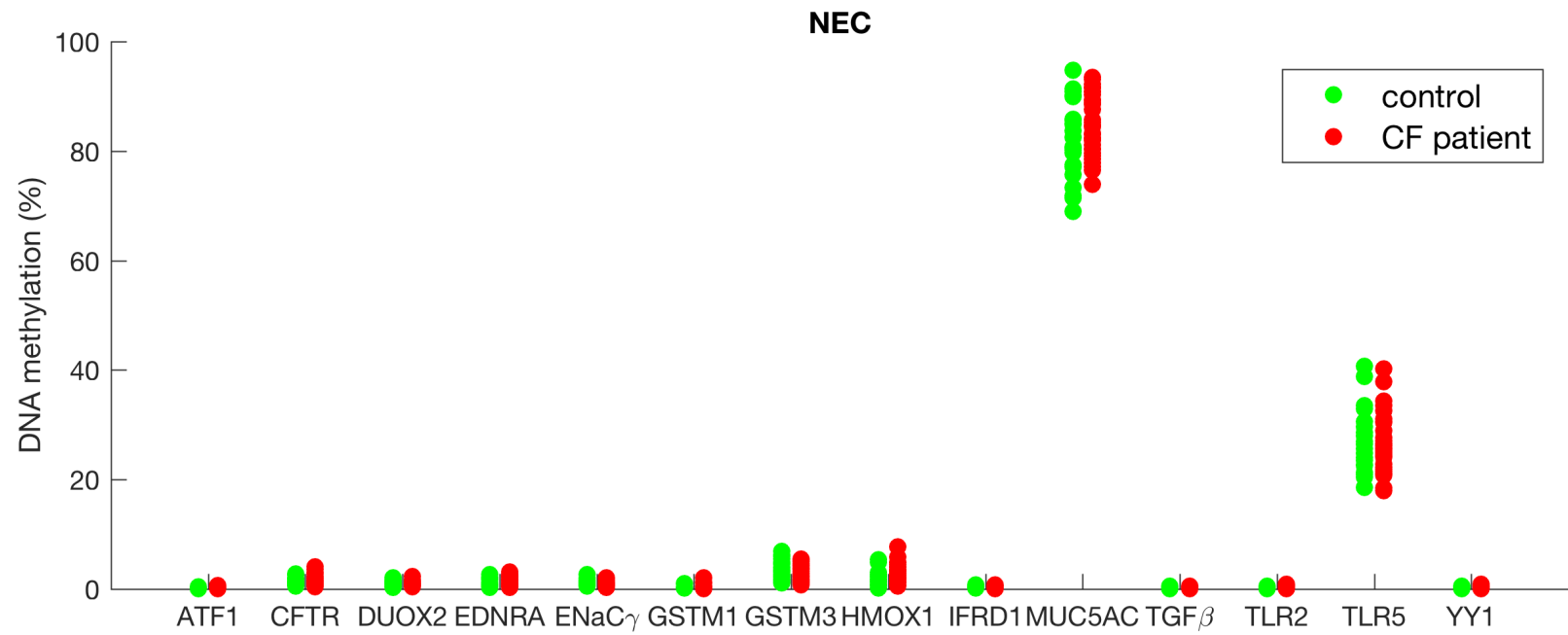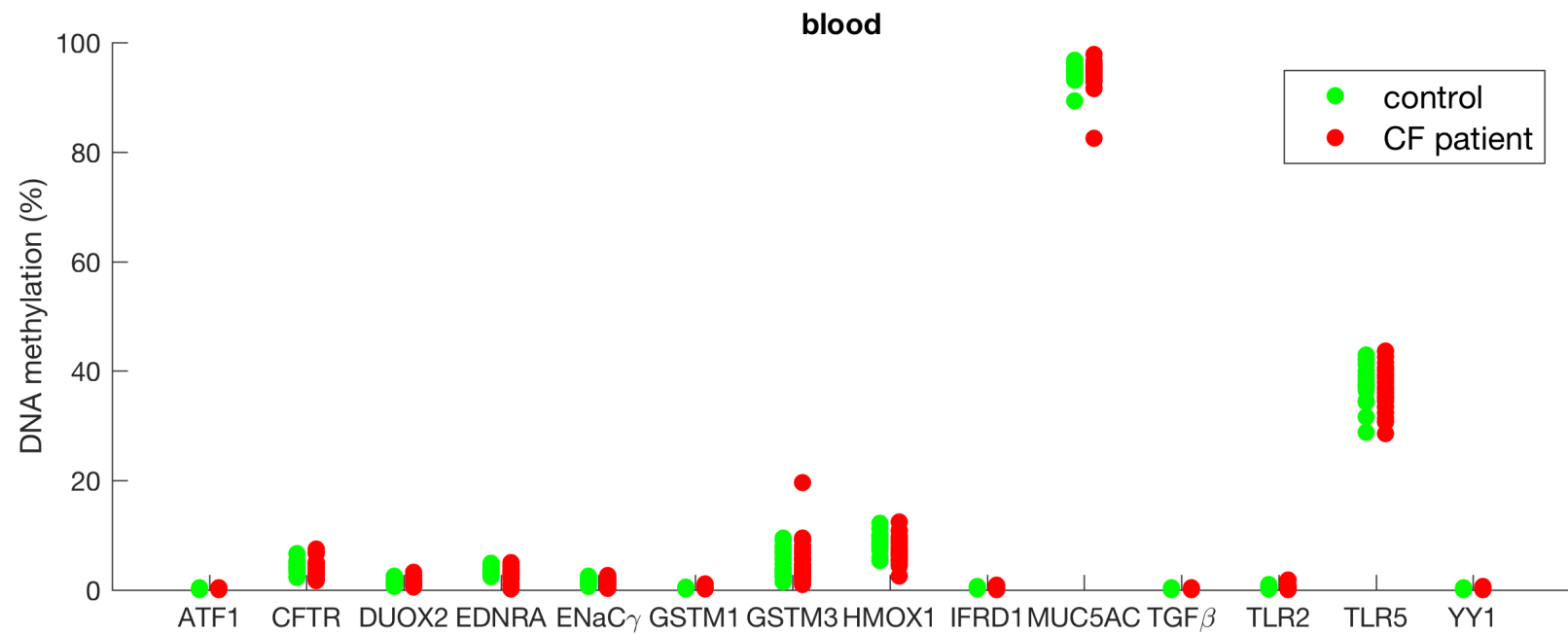

Supplement: Additional file 2: Figure S2. — DNA methylation distribution at 14 analyzed genes in CF and control samples. (PDF 212 kb) [file 13148_2016_300_MOESM2_ESM.pdf]

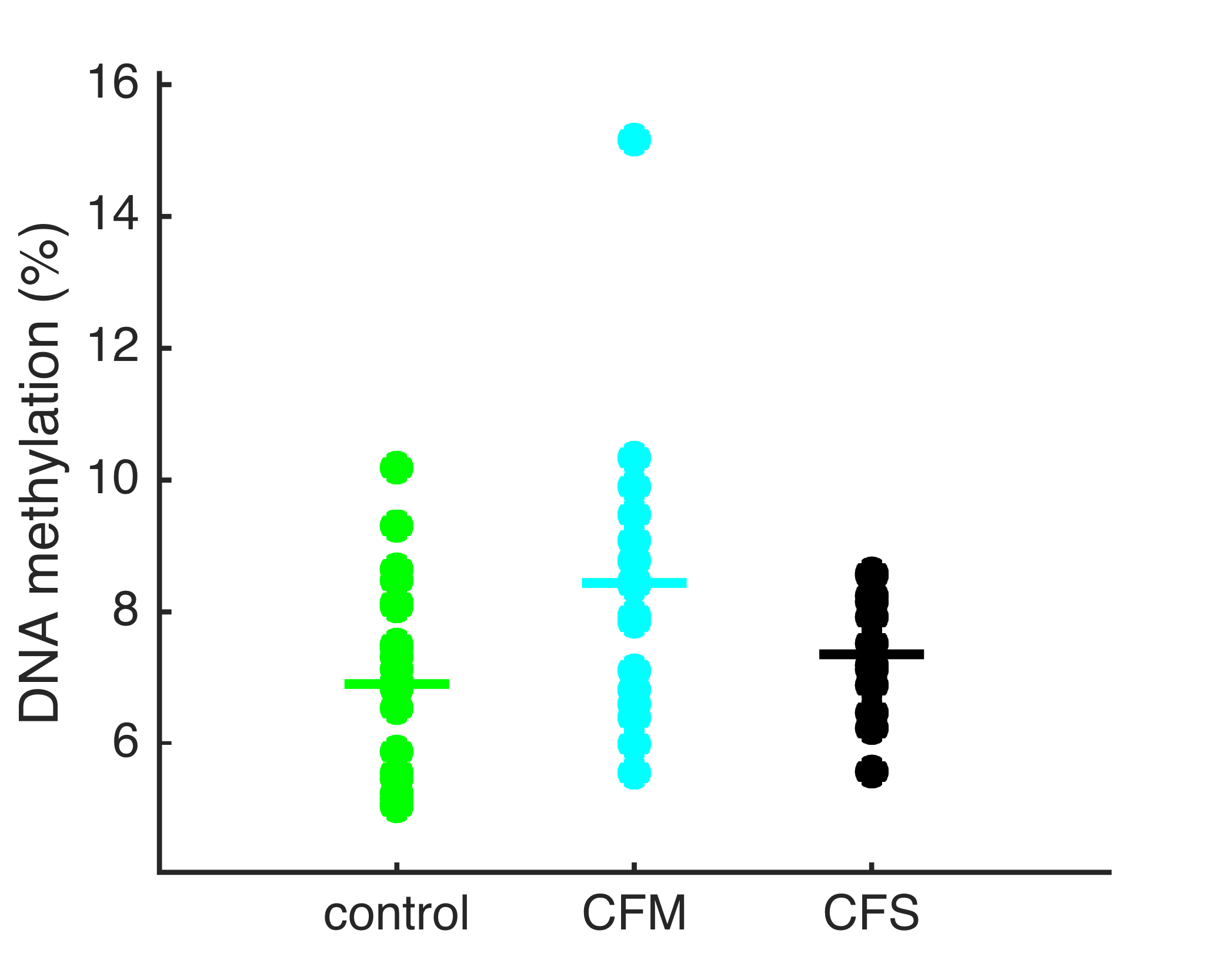

Supplement: Additional file 4: Figure S3. — DNA methylation analysis at EDNRA in an independent set of CF patients. DNA methylation at EDNRA was associated with pulmonary severity in blood samples collected from this independent set of severe (CFS) and mild (CFM) CF patients (Kruskal-Wallis p = 0.047). CF patients were from the FrGMC cohort; controls were from the METHYLCF cohort. The mean DNA methylation was measured by pyrosequencing. (PNG 72 kb) [file 13148_2016_300_MOESM4_ESM.png]
