# Supplementary material for: DNA methylation at modifier genes of lung disease severity is altered in cystic fibrosis
Source: Clin Epigenetics. 2017 Feb 14;9:19. doi: 10.1186/s13148-016-0300-8 (PMC5310067; doi:10.1186/s13148-016-0300-8)
Supplement: Additional file 3: Table S2. — Distribution of HMOX1 and GSTM3 genotypes in CF patients and controls (DOCX 39 kb) [file 13148_2016_300_MOESM3_ESM.docx]

**Table S2**. Distribution of *HMOX1* and *GSTM3* genotypes in CF patients and controls

| Gene | Genotype | Controls (n=24) | CF patients  (n=48) | p-value  (Chi-square) |
| --- | --- | --- | --- | --- |
| *HMOX1* (rs2071746) | AA | 9 | 18 | p>0.05 |
|  | A/T | 12 | 21 |  |
|  | TT | 3 | 9 |  |
|  |  |  |  |  |
| *GSTM3* | AA | 17 | 36 | p>0.05 |
|  | A/B^1^ | 4 | 11 |  |
|  | BB^1^ | 3 | 1 |  |

^1^GSTM3*B allele has a polymorphic 3-bp deletion
