# Supplementary material for: DNA methylation at modifier genes of lung disease severity is altered in cystic fibrosis
Source: Clin Epigenetics. 2017 Feb 14;9:19. doi: 10.1186/s13148-016-0300-8 (PMC5310067; doi:10.1186/s13148-016-0300-8)
Supplement: Additional file 5: Table S1. — Primers. (DOCX 49 kb) [file 13148_2016_300_MOESM5_ESM.docx]

**Table S1.** Primers

| ***BS-NGS*** | | | | |
| --- | --- | --- | --- | --- |
| Gene | | Forward primer | Reverse primer | Annealing  Temperature (°C) |
| *ATF1* | | 5'-TAGATAGTTTTGGAGAGATG-3' | 5'-CACAACCAAACATTATTTCAC-3' | 56 |
| *CFTR* | | 5'-GTTGTTAATTGGATTTAAAGAGAGG-3' | 5'-CTTTCCCCATTCTAACTCCCAACC-3' | 56 |
| *DUOX2* | | 5'-TAGGAAGTTTGTTGTTAGAG-3' | 5'-TCCAAACAACCCCAACTTAC-3' | 56 |
| *EDNRA* | | 5'-GATAGTTGGAAGGTTAGG-3' | 5'-TCCTAAACACTACTTCTCCC-3' | 56 |
| *ENaCγ* | | 5'-TAGTAATAGGGGGTAGTAGG-3' | 5'-CACCTACCCAAATACTTTCC-3' | 56 |
| *GSTM1* | | 5'-GGGAGGAAGTTTTATTGAGTGT-3' | 5'-CCAATACCCCAATATCATAAAC-3' | 56 |
| *GSTM3* | | 5'-GATTGATAGAGAGGGGAGTTTG-3' | 5'-CTTCATTCATTAATAATACC-3' | 52 |
| *HMOX1* | | 5'-GGGATATTGTTATATAG-3' | 5'-AACACTCCCCATCTTAATCAC-3' | 52 |
| *IFRD1* | | 5'-ATATTTGTATTTTTAGGGTG-3' | 5'-CACTCTCAAACCCAAAAACC-3' | 52 |
| *MUC5AC* | | 5'-GTGGAGGTGTTTTTTAGTAAGTTTG-3' | 5'-CATACCTATAACCTACACTCAAC-3' | 56 |
| *TGFβ1* | | 5'-TTGAGGTTTTAGAGTTTGAG-3' | 5'-AATCCCCAAATCCTACCTCC-3' | 56 |
| *TLR2* | | 5'-GGGAATGTGGATATTAG-3' | 5'-CAACCCATTAACACTACAC-3' | 52 |
| *TLR5* | | 5'-TGTTATGTGGGTGTTTTGTG-3' | 5'-CAAACCACCTACCTCTTCCA-3' | 58 |
| *YY1* | | 5'-TATTTTTTAGAAGGAGGGGG-3' | 5'-CAAACCCTTTCCACTAAATA-3' | 56 |
| ***Real Time PCR*** | | | | |
| *GAPDH* | 5'-CCATCTTCCAGGAGCGAG-3' | | 5'- CTTGAGGCTGTTGTCATA-3' | 60 |
| *HMOX1* | 5'-CTGAAGGAGGCCACCAA-3' | | 5'- GCTCTTCTGGGAAGTAGACA-3' | 61 |
| *EDNRA* | 5'-GCCATTCCTGAAGCGAT-3' | | 5'-GGCATACAGAAATAGAACCC-3' | 60 |
| *TBP* | 5'-CACGAACCACGGCACTGATT-3' | | 5'-TTTTCTTGCTGCCAGTCTGGAC-3' | 60 |
